# Spatial Spread of the Root Parasitic Weed *Phelipanche aegyptiaca* in Processing Tomatoes by Using Ecoinformatics and Spatial Analysis^†^

**DOI:** 10.3389/fpls.2017.00973

**Published:** 2017-06-20

**Authors:** Yafit Cohen, Itai Roei, Lior Blank, Eitan Goldshtein, Hanan Eizenberg

**Affiliations:** ^1^Institute of Agricultural Engineering, Agricultural Research Organization, Volcani CenterRishon Lezion, Israel; ^2^The Robert H. Smith Faculty of Agriculture, Food and Environment, The Hebrew University of JerusalemRehovot, Israel; ^3^Institute of Plant Protection, Agricultural Research Organization, Volcani CenterRishon Lezion, Israel; ^4^Newe Ya’ar Research Center, Agricultural Research Organization, Volcani CenterRishon Lezion, Israel

**Keywords:** Egyptian broomrape, parasitic weeds mapping, geostatistics, average nearest neighbor (ANN), multiscale analysis

## Abstract

Egyptian broomrape (*Phelipanche aegyptiaca*) is one of the main threats to tomato production in Israel. The seed bank of *P. aegyptiaca* rapidly develops and spreads in the field. Knowledge about the spatio-temporal distribution of such weeds is required in advance of emergence, as they emerge late in their life cycle when they have already caused major crop damage. The aim of this study is to reveal the effects of two major internal infestation sources: crop rotation and infestation history; and one external source: proximity to infested tomato fields; on infestation of *P. aegyptiaca* in processing tomatoes. Ecoinformatics, spatial analysis and geostatistics were used to examine these effects. A regional survey was conducted to collect data on field history from 238 tomato fields between 2000 and 2012, in a major tomato-growing region in Israel. Multivariate logistic regression in the framework of generalized linear models (GLM) has demonstrated the importance of all three variables in predicting infestation in tomato fields. The parameters of the overall model indicated a high specificity between tomatoes and *P. aegyptiaca*, which is potentially responsible for aggravating infestation. In addition, *P. aegyptiaca* infestation levels were intensively mapped in 43 of the 238 tomato fields in the years 2010–2012. Geostatistical measures showed that 40% of the fields had clustered infestation spatial patterns with infestation clusters located along the fields’ borders. Strong linear and negative relationships were found between infestation level and distance from a neighboring infested field, strengthening the role of infested tomato fields in *P. aegyptiaca* spread. An experiment specifically designed for this study showed that during harvest, *P. aegyptiaca* seeds are blown from an infested field to a distance of at least 90 m, and may initiate infestation in neighboring fields. Integrating current knowledge about the role of agricultural practices on the spread of *P. aegyptiaca* with the results of this study enabled us to propose a mechanism for the spread of *P. aegyptiaca*. Given the major effect of agricultural practices on infestation levels, it is assumed that the spread of this weed can be suppressed by implementing sanitation and using decision support tools for herbicide application.

## Introduction

The broomrapes are root parasitic plants from the genera *Orobanche* and *Phelipanche* in the *Orobanchaceae* family Broomrapes. Several broomrape species have specialized in attacking and damaging vegetables and field crops such as sunflower, oilseed rape, carrot, fava bean, and tomato in areas around the Mediterranean, in central and eastern Europe, and in Asia (Parker, 2009). In the Mediterranean areas, and specifically in Israel, the number of infested fields has increased dramatically during the last decade, causing heavy damage or even total yield losses in some places. In Israel, Egyptian broomrape (*P. aegyptiaca*) is one of the main threats to tomato production.

Control strategies designed for non-parasitic weeds do not necessarily achieve the required level of control for broomrape (Fernández-Aparicio et al., 2016). For root parasitic weeds, chemical control should be applied before shoot emergence (in the soil subsurface), because they emerge late in their life cycle when they have already caused major crop damage (Goldwasser and Kleifeld, 2004; Eizenberg et al., 2013). Therefore, knowledge of the spatio-temporal distribution of such weeds is required in advance of emergence. Several temporal models for predicting the parasitism dynamics based on thermal time have been proposed for *O. cumana* (sunflower broomrape) in sunflowers, *P. aegyptiaca* in tomatoes, *O. minor* (small broomrape) in red clovers, *O. crenata* (crenate broomrape) in fava beans and lentils, and *P. aegyptiaca* in carrots (Eizenberg et al., 2005, 2012b; Ephrath et al., 2012; Cochavi et al., 2016; Pérez-de-Luque et al., 2016). Accordingly, chemical control protocols based on thermal time models have been developed for sunflowers, processing tomatoes and carrots (Cochavi et al., 2016; Eizenberg et al., 2012a,b). One of the main drawbacks of these models was that they proposed a uniform approach for chemical applications, without taking into consideration the spatial distribution of the broomrape infestations, as has previously been shown to be relevant in parasitic weed control (Gonzalez-Andujar et al., 2001) and specifically to site-specific parasitic weed management (Eizenberg et al., 2013).

Seed banks of root parasitic weeds (broomrapes and *Striga* sp.) species develop rapidly in fields with suitable hosts, mainly due to their minute size (0.1–0.3 mm) and extreme longevity (Bebawi et al., 1984; Lopez-Granados and Garciatorres, 1993). Understanding the infestation mechanism of new fields and ways to prevent or minimize seed spread within a field should therefore be major objectives of parasitic weed management and tactics (Rubiales et al., 2009; Fernández-Aparicio et al., 2016). Very few studies have explored the dynamics of broomrape dissemination in agricultural fields and their associated variables. Instead farmers, extension workers and researchers, for the most part, assume that human practices are major significant factors responsible for the dissemination of broomrape, i.e., that they are transported by contaminated agricultural vehicles, farm implements and produce containers (e.g., Yaacoby et al., 2015; Fernández-Aparicio et al., 2016). Parasite seed distribution is also thought to be caused by the transportation of contaminated plant material (such as crop seeds and hay) and the movement of contaminated soil and manure (Goldwasser and Rodenburg, 2013; Fernández-Aparicio et al., 2016). Studies on spatial distribution and within-field spread of broomrape are quite limited. Lyra et al. (2016) demonstrated that soil and climatic variables were key explanatory factors for variations in the scope of infestation of *P. aegyptiaca* and *P. ramose* in large areas in Greece. In their study, soil pH, the content of organic matter and total soil humidity were the most decisive variables for the severity of infestation within a field. A different study investigated the dynamics of *O. crenata* parasitism on a relatively small experimental plot over a period of eight years (Lopez-Granados and Garciatorres, 1993). They showed that a minor initial infestation considerably increased after eight consecutive growing seasons of fava beans. The annual growth rate of the *O. crenata*, however, widely varied between years and was significantly correlated with rainfall and soil temperatures in certain months. Excluding the first year of the experiment, *O. crenata* populations demonstrated spatial autocorrelation, shifting from relatively clustered to dispersed patterns (Gonzalez-Andujar et al., 2001). To some extent, the spatial distributions in subsequent years were positively related, demonstrating patch-location stability (Oveisi et al., 2010). Nonetheless, no attempt was made to ascertain what factors were associated with the patches.

It has been proposed that ecoinformatics approaches, which are based on large quantities of data, should be used to address ecological questions in agricultural eco-systems at larger spatial and temporal scales than are typically feasible within an experimental framework (Rosenheim et al., 2011). Indeed, ecoinformatics approaches have been used to reveal the effect of crop rotation histories on cotton yield (Meisner and Rosenheim, 2014); the effects of local and landscape factors on pest distribution (Carrière et al., 2012; Parsa et al., 2012); and the effect of soil and bioclimatic factors on the infestation level of tobacco by species of *Phelipanche* (Lyra et al., 2016).

To the best of our knowledge, no studies were published that examined the effect of crop rotation and infestation histories on the spread of broomrape species in commercial fields. The aim of the current study is to reveal the effects of two major internal infestation sources: crop rotation and infestation history; and one external source: proximity to infested tomato fields; on infestation of the root parasitic weed *P. aegyptiaca* in processing tomatoes. Ecoinformatics, spatial analysis and geostatistics were the methods used to examine these effects. A mechanism for analyzing multi-scale spatial infestation of *P. aegyptiaca* in processing tomatoes is proposed.

## Materials and Methods

### Study Period and Area

**FIGURE 1:**
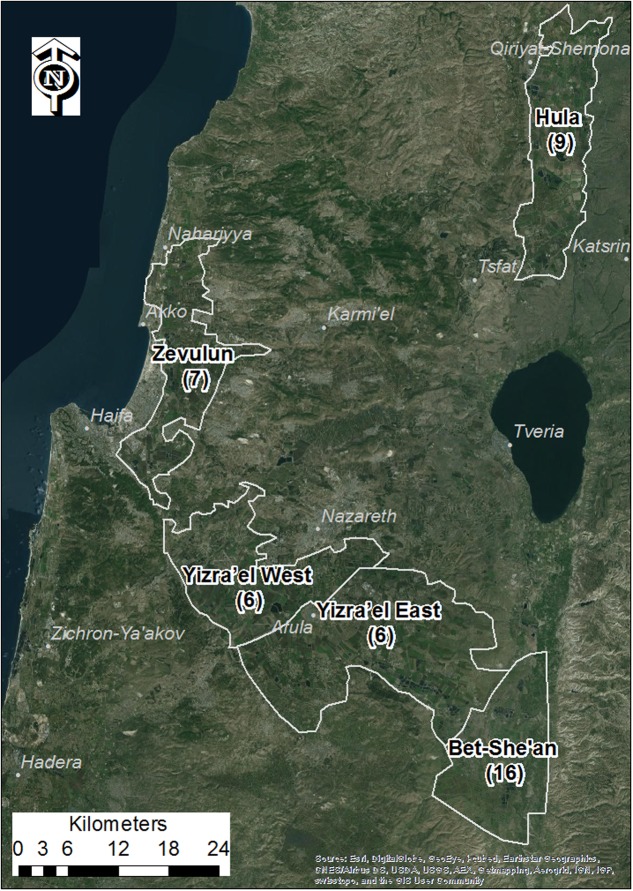
Study area valleys. Numbers in brackets are the number of densely sampled tomato fields from 2010 to 2012

Five major tomato growing regions in northern Israel were selected for mapping *P. aegyptiaca* infestation in tomato fields, from 2000 to 2012, the Bet-She’an Valley, the Western and Eastern Yizra’el Valleys, the Zevulun Valley, and the Hula Valley (**Figure 1**). The tomato growing season in all of these regions extends for about 120 days, with variations in planting and harvesting months due mainly to differences in air and soil temperatures (**Table 1**).

**Table 1:** Typical planting and harvesting months in the five tomato-growing regions in Northern Israel.

Regions	Planting	Harvesting
Bet-She’an Valley	February	June
Western and Eastern Yizra’el Valleys	March	July
Zevulun and Hula Valleys	April	August

### Surveys of *P. aegyptiaca* Infestation in Fields

Two types of surveys of *P. aegyptiaca* infestation were conducted across the five regions. Historical data from 2000 to 2012 were collected from the farmers. The farmers were asked for the following information: crop rotation, history of *P. aegyptiaca* infestation in tomatoes and in other broomrape crop hosts (year and level); *P. aegyptiaca* infestation (year and level) in neighboring tomato fields; chemical and other control applications; and historical locations of the containers. To enable the farmers to judge the infestation level, photographs of three levels (high, medium, and low) of infestation were shown to the farmers (Eizenberg et al., 2012a). Despite our efforts to ensure that the data collection was as complete as possible, we were reliant on the farmers’ records and recollections. Therefore, the data set compiled from the surveys contained only partial data, with the historical data collected from the farmers on broomrape infestation and/or crop rotations comprising a total of 238 records. The other data types (i.e., chemical and other control applications, and historical locations of the containers) were available for a much smaller number of tomato fields and were not used for further analysis.

The second type of survey was conducted in the years 2010–2012, and consisted of mapping the within-field infestation distribution in tomato fields. Infestation was sampled in 43 fields, in a systematic rectangle grid pattern with a sampling density of 42 samples/hectare. Sampling was conducted in every 12th crop row (2 m wide), which was divided into fixed 10 m intervals. In this way, each sample represented an area of 240 m^2^ (24 m × 10 m). Based on shoot emergence, each unit sample (240 m^2^) was categorized into one of four infestation levels:

(0) No shoots: no infestation.
(1) 1–50 shoots per unit sample (equivalent to <2 shoots per 10 m^2^): low infestation level.,
(2) 50–200 shoots per unit sample (2–8 shoots 10 m^2^): medium infestation level,
(3) More than 200 shoots per unit sample (>8 shoots 10 m^2^): high infestation level.

**FIGURE 2:**
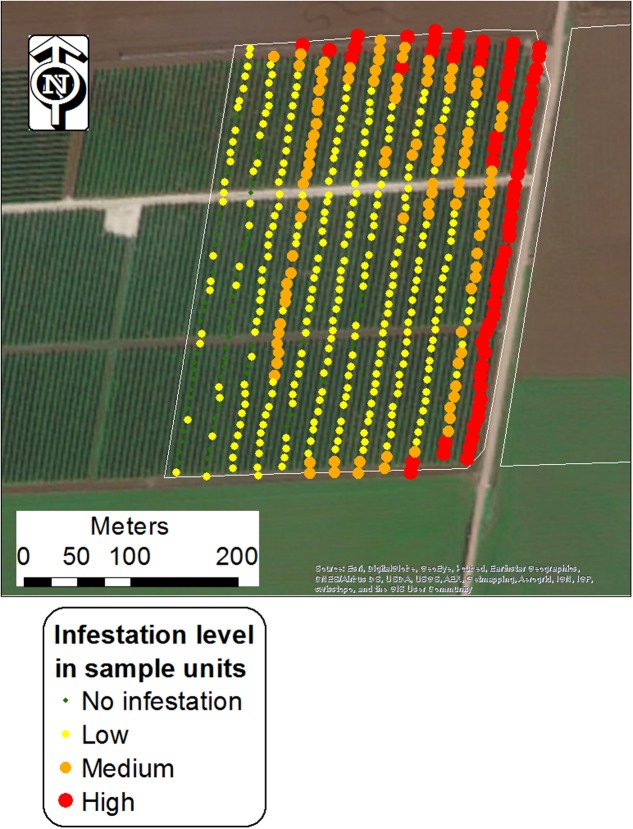
Central points of unit samples in a tomato field (Kishon 1; West Yizra’el Valley region) grouped into four infestation levels. Each point represents an area of 240 m^2^ (24 m × 10 m).

Each unit sample was visually scanned and assigned to the different infestation categories based on estimations. Sampling was conducted using a mobile GPS-GIS (MobileMapper 10, Ashtech LLC, with real-time satellite-based augmentation system [SBAS] typically < 2 m) at the end of the tomato biological cycle (June to August), when broomrape populations were thriving and could be easily detected. **Figure 2** shows one of the surveyed fields with the locations of the sampling points, grouped into the four infestation levels.

The data collected from the whole-field-scale and from the within-field-scale were structured and stored in geodatabases to enable effective multi-year data updating, data processing, and analysis using ArcGIS 10 software (ESRI, Ltd). The two databases were combined, to enable us to postulate relationships between data from the two scales and to use their complementarity to characterize the spread dynamics of *P. aegyptiaca*.

### Statistical Analysis

The influence of two major internal infestation sources: crop rotation and infestation history; and one external source: proximity to infested tomato fields, on *P. aegyptiaca* infestation was estimated by logistic regression in the framework of generalized linear models [GLM]. The explanatory categorical variables are listed in **Table 2** with their categories.

**Table 2:** Explanatory variables included in the generalized linear models (GLM).

Variable description	Category abbreviation	Category description
Infestation history	NoINF	Fields that were not parasitized by *P. aegyptiaca* in the past (*P. aegyptiaca* host free)
	INFinTomato	Fields that were parasitized by *P. aegyptiaca* in the past, in tomato crops
	INFinOHost	Fields that were parasitized by *P. aegyptiaca* in the past in hosts other than tomatoes^‡^
	INFinBoth	Fields that were parasitized by *P. aegyptiaca* in the past in both tomatoes and in other hosts
Crop rotation	CRnoHosts	Crop rotation that does not include any *P. aegyptiaca* host (*P. aegyptiaca* host free)
	CRwithTomato	Crop rotation that includes tomato crops
	CRwithOHost	Crop rotation that includes *P. aegyptiaca* hosts other than tomatoes
	CRwithBoth	Crop rotation that includes both tomato crops and other *P. aegyptiaca* hosts
Neighboring field	NoNeigh	Fields with no proximity to infested neighboring tomato fields ^l^
	NeighINFinTomato	Fields with proximity to infested neighboring tomato fields

^‡^*Hosts other than tomatoes include sunflowers, legume crops like vetch and chickpea and umbel crops like carrot and parsley.*

^l^*Neighboring infested tomato field was defined where it was parasitized by *P. aegyptiaca* in the past with medium to high infestation levels*.

We used logistic regression in the framework of GLM to relate the explanatory variables to the infestation level. Multi-model inference based on the Akaike Information Criterion [AIC] was used to rank the importance of variables (Burnham and Anderson, 2002; Saltz, 2011; Blank and Blaustein, 2014). The AIC is increasingly being used for measuring and ranking competing models by evaluating the goodness of fit and the number of used variables in each model. Models with fewer variables will be favored. The model having the lowest AIC value represents the best approximating model. The coefficients associated with each variable and their relative importance were assessed using a multi-model average. Using categorical variables, the regression model creates dummy variables for k-1 categories for each variable, where k is the number of categories in each variable. The remaining category for each variable is used as the reference level for the other categories in that variable. In our case, the categories NoINF for infestation history, CRnoHosts for crop rotation and NoNeigh for neighboring field were used as the reference levels. Based on the dummy variables, estimates are calculated for the k-1 categories for each variable, and additional estimate calculated for the intercept, which is a common estimate for the reference categories. The associated p-values are for the tests of the indicated category vs. the reference level in isolation. To determine the predictor estimates, we calculated the unconditional variance and the confidence intervals (95% CI). All statistical analyses were carried out with R 3.1.0 (R Core Development Team).

### Spatial Pattern Analysis

Point pattern analysis can be used to detect the spatial arrangement of infestation and to generate hypotheses as to the possible underlying factors and/or processes controlling the observed pattern. The nearest-neighbor distance geostatistic (Clark and Evans, 1954) was applied, using the average nearest neighbor [ANN] tool (ArcGIS 10.3.3, ESRI, Ltd.) to detect whether a point pattern of infestation departed from an assumed random Poisson point pattern. The ANN tool was applied to every sampled field (43 fields) using the sampling points with an infestation level higher than 1. The ANN tool calculated the distance between the location of each sampling point and the location of its nearest neighbor’s point; and the average of all these nearest neighbor distances. If the average distance was significantly lower than the average for a hypothetical random distribution, the infestation distribution was considered clustered. If the average distance was greater than a hypothetical random distribution, the infestation distribution was considered dispersed or uniform. Otherwise, it was considered random as the null hypothesis.

### Spatial Analysis

**FIGURE 3:**
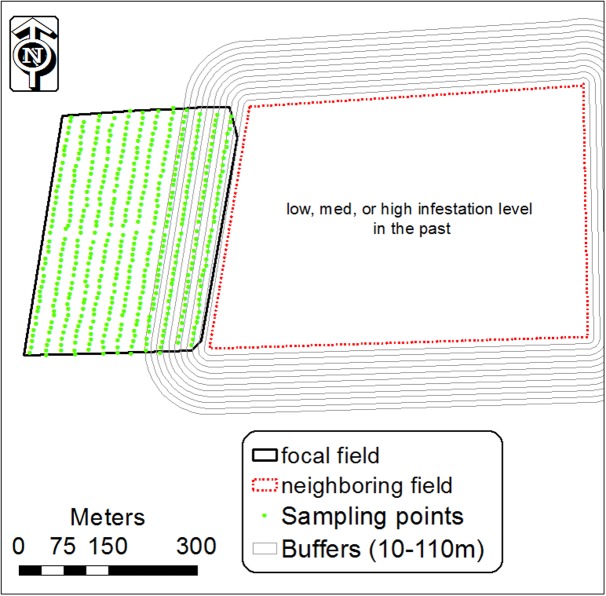
Buffers of 10–110 m around an infested tomato field designated the focal field, and infestation sampling center points in the neighboring tomato field. Inside each buffer, the mean level of infestation was calculated from the center points inside it.

The results of the GLM analysis and infestation spatial patterns indicated that infested fields are a possible contributor to the initial appearance of *P. aegyptiaca* in neighboring fields. To further explore this conjecture, 11 buffers (1, 10, 20, 30, 40, 50, 70, 90, 110 m) were built from the boundary of the infested field, designated the focal field, into the neighboring field (**Figure 3**). For each buffer, the average infestation level was calculated on the basis of the infestation level of the sampling center points that fell inside it, using the spatial join module (ArcGIS 10). We then calculated the regression between the average infestation level inside each buffer and the buffer distance from the field boundary. This was done for different tomato field groups, grouped by crop rotation (with or without tomato), infestation history (were or were not infested in the past) and infestation level.

### A Field Experiment

An experiment was conducted to detect the dispersal mechanism of *P. aegyptiaca* seeds from one field to its neighbor. The assumption was that the *P. aegyptiaca* seeds are blown into the adjacent field during the tomato harvest by the blower of the combine harvester. During the 2012 harvest period, two highly infested tomato fields were selected for this experiment, one in the Bet-She’an Valley (denoted HEden) and the other in the Hula Valley (HGadash). Plastic cards (16 cm × 16 cm) were smeared with insect glue and attached to 10 cm high poles and placed at distances of 20, 50, and 90 m from the fields’ borders in adjacent bare fields. During that growing season, no *P. aegyptiaca* hosts were grown in those fields to ensure that seeds attached to the cards would be external. The distances were determined on the basis of the results of the spatial analysis described in the previous section, which showed a gradual decrease in infestation level with increasing distances, which leveled off at around 100 m. Three to five cards were placed along each border of the fields at each of the three distances. On the western border of HGadash, corn was grown, and therefore cards could not be placed there properly for the experiment. Overall, 42 cards were placed around each of the fields. The HEden field was harvested on June 20–21, 2012, and HGadash was harvested on August 5, 2012. The sticky cards were put in place immediately before the harvest and removed immediately after it. As soon as they were removed, the cards were wrapped with plastic wrap to preserve the seeds that were attached to it. The cards were examined under a binocular microscope and the number of attached *P. aegyptiaca* seeds was counted. In addition, meteorological data were collected during harvest, to examine the supplementary effect of wind on movement of the seeds.

### Conceptual Simulation Model of the Spread of *P. aegyptiaca*

According to the proposed mechanism, a conceptual simulation model was developed to illustrate the *P. aegyptiaca* spread, which started with *P. aegyptiaca* introduction into a new tomato growing region, followed by its spread in the region over a period of ten years (2010–2020). The model entails a virtual tomato growing region comprised of nine fields in which a tomato crop is grown every five years (the crop rotation normally used in Israel). The model referred to three spread phases, where each phase is comprised of three consecutive growing seasons (2010–2012; 2014–2016; 2018–2020). In the model, a tomato crop was grown in the region for the first time in 2010. That year, three tomato fields with simulated initial low infestation levels were introduced. To enable within-field differences in infestation levels, every field was divided into a net of points in a regular grid pattern (similar to the grid presented in **Figure 2**), such that every point could have an individually assigned infestation level. Initial infestation was allocated in proximity to the fields’ borders or along cultivation rows, following both the findings of the spatial pattern analysis of the current study, Yaacoby et al. (2015) and Fernández-Aparicio et al. (2016). Both of the latter studies indicated that among other factors, dissemination of broomrape occurs via contaminated agricultural vehicles and produce containers. From the second year on, the impact of neighboring infested fields was added wherever applicable, according to the results of the current study. In the second and the third phases, where fields were planted with tomatoes for the second and the third time, the impact of the internal infestation source was added. On average, the infestation level of the points inside each field increased by one every time tomato was grown for the second and the third time in that field. The magnitude of one was defined following the calculation of the average differences in infestation levels between successive tomato growing seasons of real fields in the historical database. The average difference between infestation levels between the second and the first tomato crop was 0.89 (*n* = 106) and between the third and the second tomato crop was 0.97 (*n* = 11). In addition, the possibility of relative stability in infestation patches (Oveisi et al., 2010) was taken into account, leading to a gradual expansion of patches.

## Results

### Spatio-Temporal Change in Regional Infestation

**FIGURE 4:**
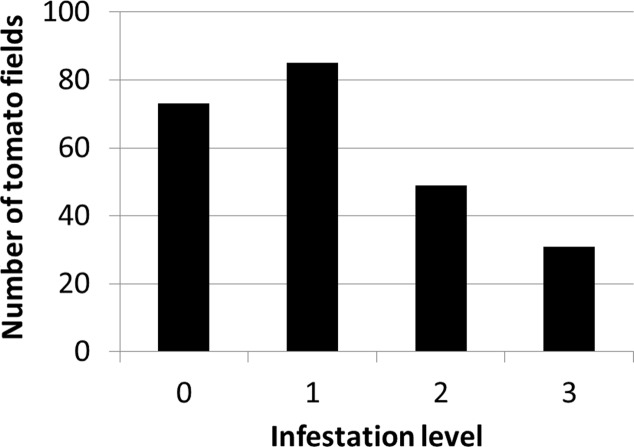
Distribution of infestation levels in all tomato fields between 2000 and 2012 (*n* = 238)

**FIGURE 5:**
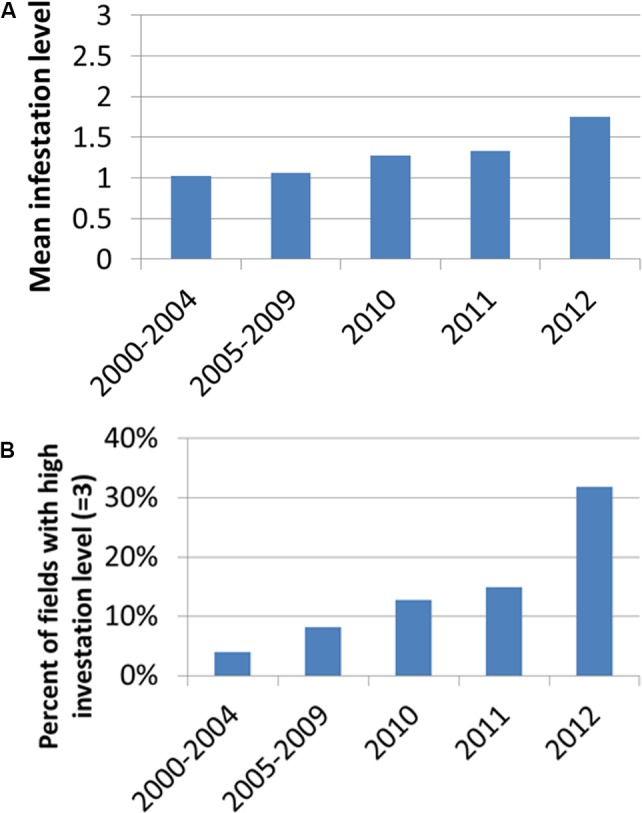
Mean infestation level **(A)** and percentage of fields with high infestation levels (level 3) **(B)** for the years 2000–2012. Because only a few records were available from 2000 to 2009, we divided the data in this period into two groups of 5 years: 2000–2004 (22 tomato fields) and 2005–2009 (43 tomato fields).

**Figure 4** presents the distribution of infestation levels in all tomato fields in the database (*n* = 238). The tomato fields had an average infestation level of 1.26 ± 0.96. **Figure 5** presents the change in mean infestation level over the years. Because only a few records were available from 2000 to 2009, we divided the data in this period into two groups of five years each: 2000–2004 (22 tomato fields) and 2005–2009 (43 tomato fields). A gradual increase in mean infestation level is evident in **Figure 5A**. The infestation level was constantly low (level = 1) in the 2000s, followed by an increase of 30%–75% at the beginning of the 2010s. The increased infestation over the years was accompanied by an accelerated rate of high infestation levels in the fields examined (**Figure 5B**). While during the 2000s, only 5% of the fields were highly infested, by 2012 more than 30% of the fields were highly infested (**Figure 5B**).

**FIGURE 6:**
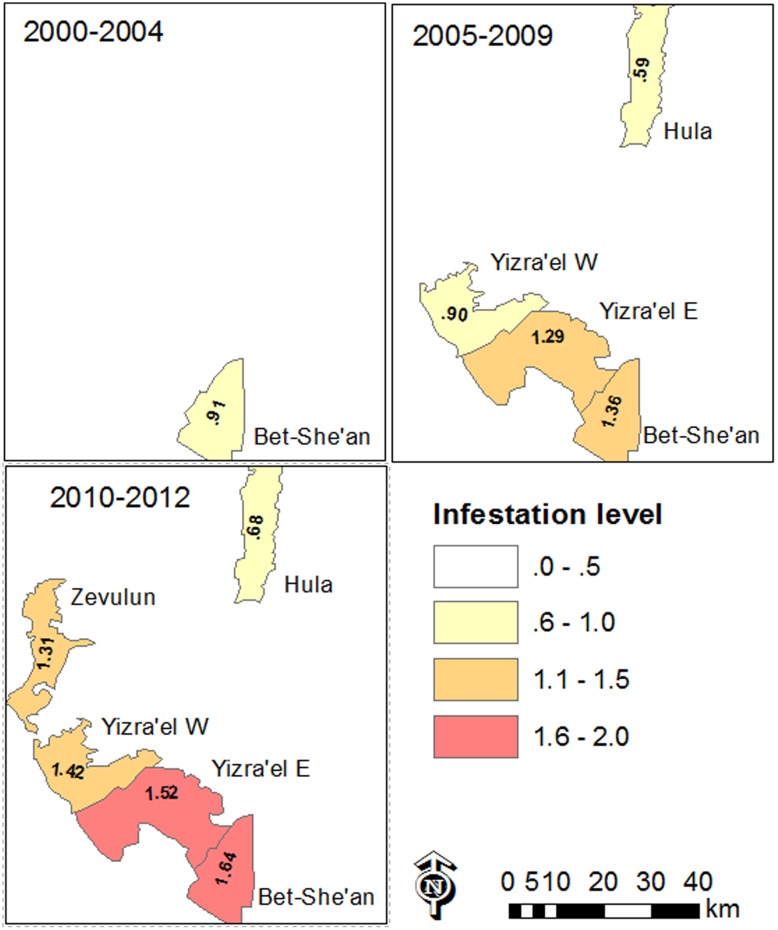
Map of mean infestation level in the various valleys in the study area over different periods of time between 2000 and 2012. Infestation level is shown only where data was available to at least ten tomato fields.

The data also revealed differences between the regions. **Figure 6** shows the change over time and space between 2000 and 2012. In general, in most of the study regions, infestation levels increased throughout the decade of the 2000s. In the Bet-She’an Valley, where sufficient data was available from 2000, the infestation levels increased from low to medium in about ten years. Interestingly, the infestation in the Hula valley was significantly lower than that of the other regions (Kruskal–Wallis test followed by the Steel–Dwass test; *p* < 0.05), presumably because processing tomatoes is a new crop in this specific area.

Infestation level is shown only where data was available for at least ten tomato fields.

### Factors Influencing Aggravation and Spread of Infestation

**Table 3** shows the results of the GLM analysis. Overall, eight models were created. The best model included all three variables and had a much lower AIC than the other models. The three best models included both internal and external infestation sources. Single variable models indicate that the variable that had the greatest impact is the infestation history (internal source); the second is proximity to an infested tomato field (external source); and the third is crop rotation (internal source).

**Table 3:** Summary of the GLM analysis examining the predictors of infestation.

Model			AICc
Infestation history	Crop rotation	Neighboring field	180.97
Infestation history	Neighboring field		204.84
Crop rotation	Neighboring field		241.05
Infestation history	Crop rotation		279.45
Infestation history			311.80
Neighboring field			321.86
Crop rotation			452.79
Intercept			570.02

**Table 4** shows the estimated coefficients and the variance of each category across all fitted GLM models. All categories had an importance of one but their estimates varied. According to the model, in comparison to a history of no infestation (NoINF – reference level for the categorical variables), tomato fields with a history of infestation would have a considerably higher infestation regardless of the *P. aegyptiaca* host. It is exemplified by two extreme cases: A predicted infestation level of 0.74 (i.e., the estimate of the intercept) would be calculated for a tomato field with no hosts in its crop rotation (a category which also entails no infestation history) and which is not adjacent to an infested tomato field. In comparison, a predicted infestation level of 2.7 (0.74+0.31+0.53+1.12) would be calculated for a tomato field with a history of infestation in tomatoes (INFinTomato), with crop rotation of both tomatoes and other hosts (CRwithBoth) and has an infested neighboring tomato field (NeighINFinTomato). The estimate of infestation in tomatoes is higher than the estimate of the infestation in hosts other than tomatoes alone (**Table 4**). Interestingly, there are contradicting trends in crop rotation that included *P. aegyptiaca* hosts. In comparison to a crop rotation that did not include hosts, a crop rotation with tomato and with other hosts had higher and lower predicted infestation, respectively. These estimates correspond to the average infestation levels of tomato fields in each category (2.18 and 0.93, **Table 4**). The estimate of the infestation level for a field in proximity to an infested tomato field was higher by 0.31 than that of a field that was not adjacent to an infested tomato field; this difference is much lower than the estimate of infestation history. This corroborates the hypothesis that the external source has less of an influence on infestation than does an internal source.

**Table 4:** Parameter estimates weight-averaged across all fitted GLM models, predicting infestation level in tomato fields and the average infestation level of tomato fields for each category.

Category abbreviation^‡^	Estimate	95% CI		Uncon-ditional variance	Average infestation level of tomato fields for each category in the database (±SD)
		Lower	Upper		
INFinTomato	1.12^∗^	0.64	1.60	0.06	2.40 ± 0.84
INFinOHost	0.81^∗^	0.28	1.34	0.07	1.44 ± 0.93
INFinBoth	1.23^∗^	0.06	2.39	0.35	2.5 ± 0.71
CRwithTomato	0.53	–0.18	1.23	0.13	2.18 ± 1.08
CRwithOHost	–0.45	–1.04	0.15	0.09	0.93 ± 0.73
CRwithBoth	0.53	–0.08	1.15	0.10	2.00 ± 0.94
NeighINFinTomato	0.31	–0.14	0.75	0.05	2.17 ± 0.87
Intercept	0.74	0.30	1.18	0.05	
*NoINF*	*Reference level for infestation history*				*0.80 ± 0.72*
*CRnoHosts*	*Reference level for crop rotation*				*0.88 ± 0.75*
*NoNeigh*	*Reference level for neighboring field*				*1.05 ± 1.17*

^‡^*Detailed descriptions of the variables are given in **Table 2**; ^∗^ estimate is different than 0, α < 0.05*

### Infestation Spatial Patterns in the Within-Field Scale

**FIGURE 7:**
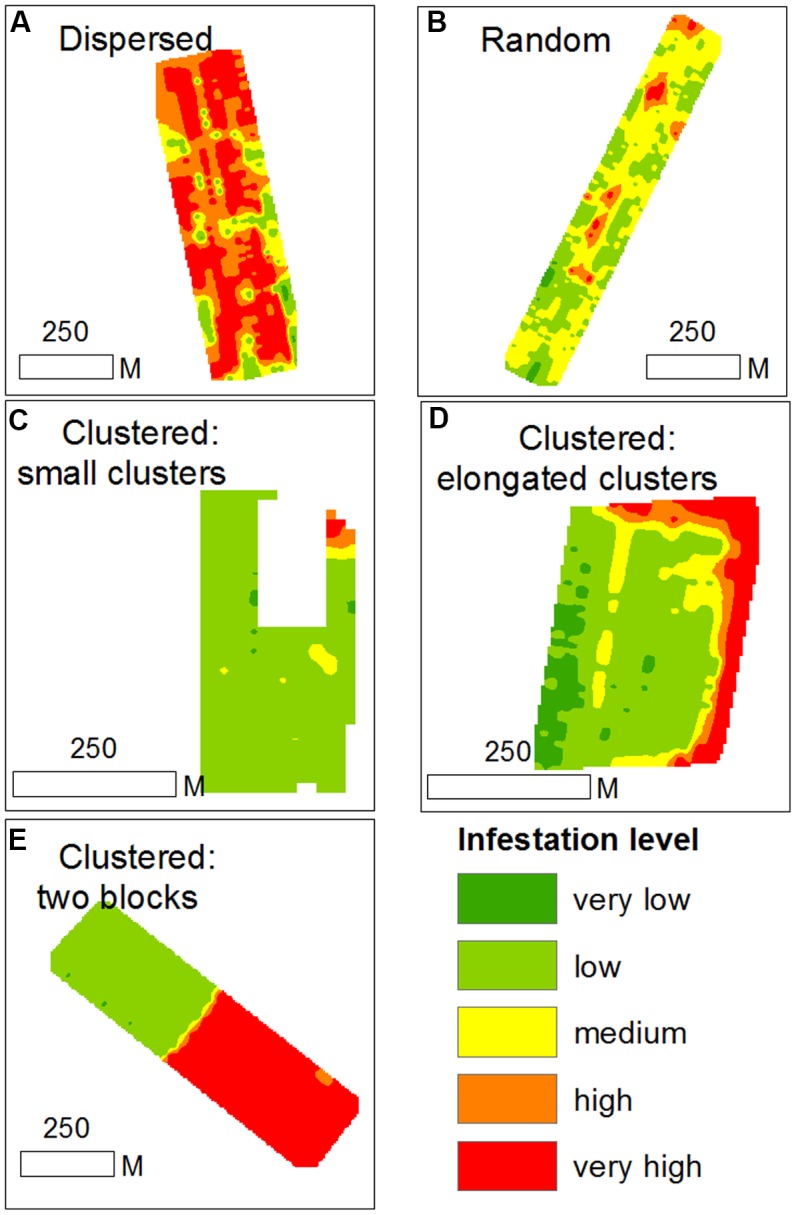
Examples of infestation spatial patterns in sampled tomato fields; **(A)** dispersed pattern; **(B)** random pattern; **(C)** clustered pattern with small clusters; **(D)** clustered pattern with elongated clusters; **(E)** cluster pattern with two distinct blocks.

**FIGURE 8:**
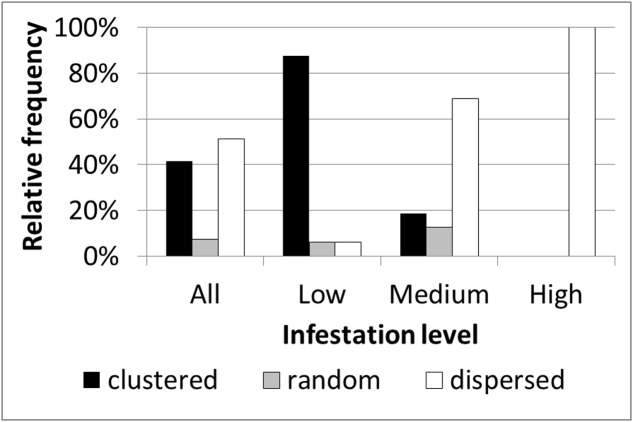
Relative frequency distribution of infestation spatial patterns according to mean infestation levels.

Based on within-field density sampling in 43 fields, three spatial patterns of infestation levels were found: random, dispersed and clustered. By visual interpretation of the spatial distributions found in the fields, the clustered patterns were further subdivided into small clusters, elongated clusters and two-block clusters (**Figure 7**). Most of the fields had dispersed and clustered patterns, while only three fields had random patterns (**Figure 8**). The relative frequency distribution of the field patterns according to mean infestation levels revealed that most of the fields with a low infestation level (88%) had a clustered pattern. By contrast, most of the fields with a medium infestation level (69%) and all the fields with a high infestation level (100%) had a dispersed pattern. Focusing on the fields with a clustered pattern (*n* = 17) exposed two main shapes of clustering: small round or elliptical shapes (**Figure 7C**, *n* = 7) and elongated shapes (**Figure 7D**, *n* = 8). Additionally, in most of these fields (*n* = 15), clusters of high infestation levels were adjacent to the borders of the fields. Each of the two remaining fields had two distinct blocks: one with high infestation and the other with no infestation or a low level of infestation (**Figure 7**). Examination of their crop rotation history revealed that the highly infested areas had tomatoes in their crop rotation and the other areas had no tomatoes in the crop rotation.

### Effect of Neighboring Infested Tomato Field

**FIGURE 9:**
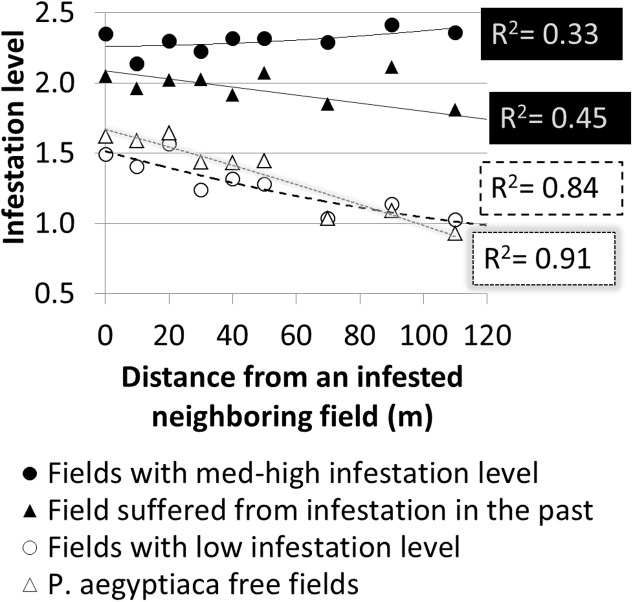
Infestation level at sampling points versus distance from borders of infested neighboring field according to infestation level and infestation history.

Further spatial analysis was conducted to examine the effect of an infested neighboring field. Based on the intensive sampling in tomato fields conducted in the years 2010–2012, the change in infestation level over the distance (0–110 m) from the borders of a neighboring infested field was calculated for fields categorized by their infestation levels or their infestation history (**Figure 9**). Strong linear relationships were found for fields with either a low infestation level (14 fields; *R*^2^ = 0.84, *p* < 0.05, *n* = 9 pairs of distances – infestation levels) or free of *P. aegyptiaca* (6 fields; *R*^2^ = 0.91, *p* < 0.05). The majority of these fields had a clustered pattern of medium to high infestation levels with elongated shapes along the fields’ borders (e.g., **Figure 7D**). Fields with either medium to high infestation levels or fields that suffered from infestation in the past had high and relatively stable infestation levels at all distances. These results correspond to the dispersed pattern that characterized fields with medium to high infestation levels (**Figure 8**).

**FIGURE 10:**
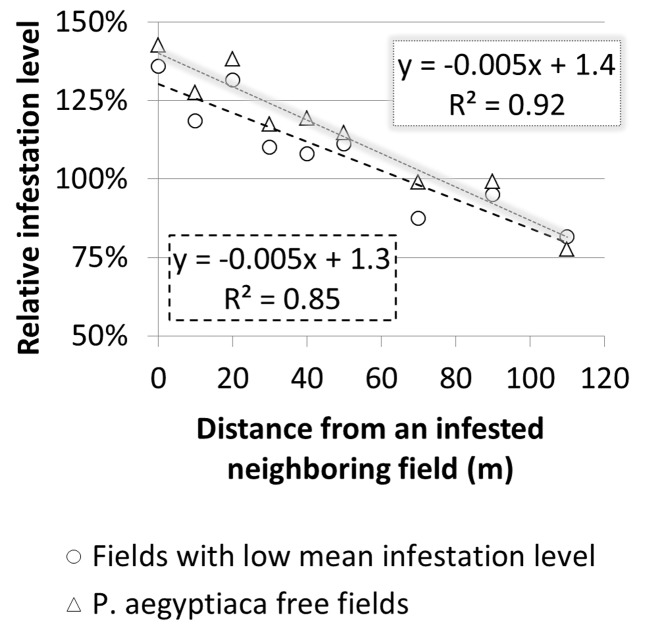
Relative infestation level at sampling points versus distance from borders of infested neighboring field in fields with a low infestation level and in *Phelipanche aegyptiaca*-free fields

To estimate the magnitude of the effect that a neighboring infested field has on the infestation of the focal field’s borders, relative infestation levels were calculated for each distance from the border, by dividing the infestation level at each distance by the overall mean infestation level of the focal field (**Figure 10**). For both groups, relative infestation adjacent to the infested neighboring field was higher by 50% than the mean infestation level of the whole field. In addition, the relative infestation decreased linearly to approximately 25% less than the mean infestation level at 110 m away from the field’s borders. The linear regression slopes (0.005) suggest that in both cases, the infestation level decreases by 5% for every 10 m from the borders.

### Spread of *P. aegyptiaca* Seeds between Fields

**FIGURE 11:**
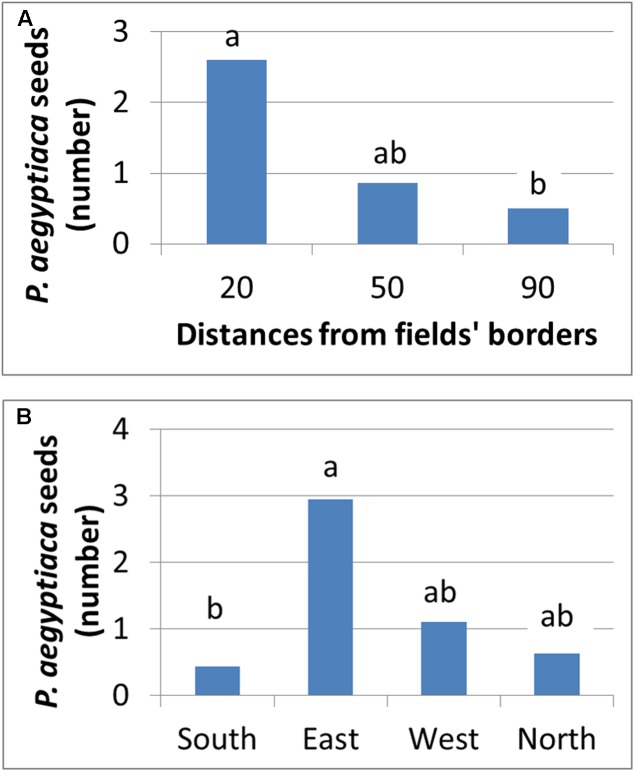
Number of *P. aegyptiaca* seeds that were attached to sticky cards at different distances **(A)** and at the four cardinal points of the compass **(B)**.

**Figure 11** presents the number of *P. aegyptiaca* seeds found on the sticky cards placed at different distances and directions from the infested field’s borders. Seeds were found at all distances, but their number decreased exponentially from 20 to 90 m as the distance from the field’s borders increased. This trend characterized both fields at the four cardinal points of the compass. At both harvest times, the major wind directions were western and northwestern. Although *P. aegyptiaca* seeds were found in all directions, more were found on the east side of the field with the sticky cards (not significant). These results indicate that during harvest, the harvester can actively blow *P. aegyptiaca* seeds to a distance of at least 90 m from the harvested fields’ borders, with the wind supplementing the effect.

### Spatio-Temporal *P. aegyptiaca* Spread in a Virtual Tomato Growing Region

**FIGURE 12:**
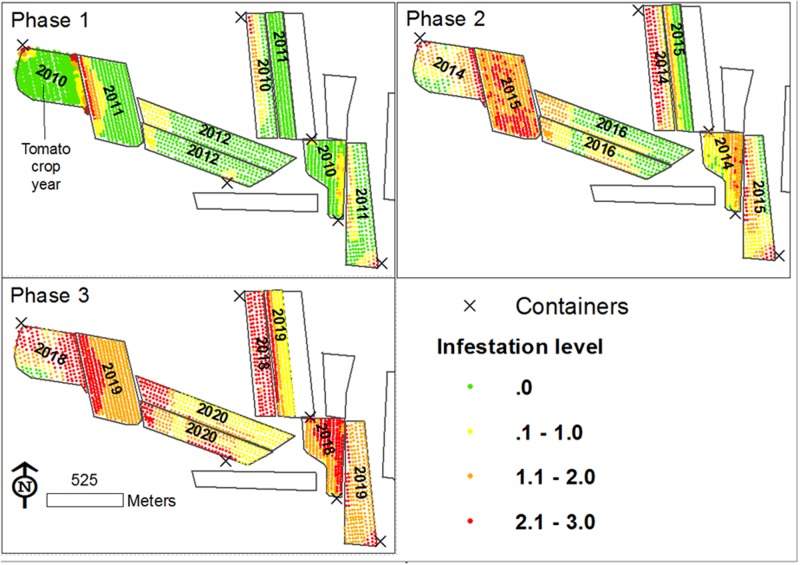
Proposed *P. aegyptiaca* spread process in tomato fields.

A foundation for defining a mechanism for the spread of *P. aegyptiaca* in tomato fields was set by combining the data scales and analysis methodologies used to study factors influencing the spread of *P. aegyptiaca*, together with knowledge from other studies. The various results indicate that both external and internal infestation sources are involved. External sources are many (Goldwasser and Rodenburg, 2013; Yaacoby et al., 2015; Fernández-Aparicio et al., 2016) and are responsible for the initial infestation. The internal source is the seed bank in the field, which is responsible for the spread in a field. Based on the proposed mechanism, a conceptual model was developed to illustrate the spread. The model started with the introduction of *P. aegyptiaca* into a new tomato growing region, followed by its spread in the region over a period of ten years. The results of the simulation are presented in three major phases, assuming crop rotation with a tomato crop every 5 years (**Figure 12**). Each phase comprises a period of three growing seasons (years):

(1) 2010–2012: Fields are planted with tomatoes for the first time;
(2) 2014–2016: Fields are planted with tomatoes for the second time;
(3) 2018–2020: Fields are planted with tomatoes for the third time.

#### The First Phase

In 2010 (the first year of the first phase) three fields were planted with tomatoes for the first time in a specific region. *P. aegyptiaca* was introduced into the fields, apparently by contaminated machinery and containers (Yaacoby et al., 2015; Fernández-Aparicio et al., 2016) which had been used in an adjacent infested region, resulting in an average low infestation level, with clustered spatial patterns (small round or elongated shapes along rows). During the tomato harvest, some *P. aegyptiaca* seeds were blown into neighboring fields. In 2011 and 2012, new fields were planted with tomatoes for the first time. *P. aegyptiaca* was introduced by contaminated machinery. In addition, because these new fields have a common border with fields infested in 2011, they contained *P. aegyptiaca* seeds that had been blown in from the infested neighboring fields during harvest. Similar to the 2010 fields, the tomato fields in 2011 and 2012 had an average low infestation level with clustered spatial patterns (mainly elongated shapes along the fields’ borders).

#### The Second Phase

In 2014–2016, tomatoes were grown for the second time. Since all the fields had internal seed banks as sources of infestation, they suffered from medium to high infestations, with random or dispersed patterns. The rapid aggravation that was presented in the simulation is in keeping with the significant effect that was found for crop rotation and infestation history, even with only one tomato crop in the infestation spread. Infested at medium to high levels, these fields became a major infestation source for their neighboring fields. Accordingly, at the end of this period, almost all the fields in the region had internal seed banks. Some had large seed banks because of their history, and some had small seed banks that originated from adjacent fields.

#### The Third Phase

In 2018–2020, tomatoes will be grown for the third time. At the end of 11 years, some fields will have three tomato crops in their crop rotation. All of these fields would have internal seed banks, and all would probably suffer from medium to high infestation levels with dispersed patterns. Moreover, neighboring fields would contain significant seed banks and if tomatoes were to be grown on them, they would probably be infested.

## Discussion

Weeds in arable lands have varied spatial patterns. Autotrophic plants emerge and can be detected when optimal conditions, e.g., soil temperature and water content exist. In contrast to the conditions required for the germination of autotrophic plants, root parasitic weeds germinate and attach to their host roots only after they are exposed to a specific germination stimulant that is exuded from the host’s roots. In most cases, root parasites emerge late in their life cycle, when they have already caused crop damage (Joel et al., 2011). If the root parasitic weed *P. aegyptiaca* is to be controlled, knowledge about its spatial and temporal distribution is required, so as to enable the implementation of control measures before the crop is damaged. In the current study, factors affecting the spatial spread of *P. aegyptiaca* in tomato fields were studied using ecoinformatics, spatial analysis and geostatistics based on a large database.

In the first stage, each data scale analyzed with the methodology relevant to that scale either provided new insights or validated a commonly held notion about the spread.

Historical data collected from farmers at the field scale quantitatively verified the major adverse effect of the seed bank in the field on infestation in the subsequent tomato crop. The common assumption among farmers, extension workers and researchers was that the source of the seed bank was irrelevant, i.e. infestation would be worse in the subsequent tomato crop even if the infestation occurred in *P. aegyptiaca* hosts other than tomato. However, historical data of tens of fields surveyed in this study strongly suggests that aggravation will most probably occur only if the source of the seed bank is *P. aegyptiaca* plants that were grown on tomatoes. This phenomenon is possibly associated with the host specificity of the parasite. Two observations made during the course of this study supported this hypothesis regarding the high specificity of the local *P. aegyptiaca* for tomatoes. One of the intensively sampled fields was heavily infested by *P. aegyptiaca* in 2010, but in the subsequent year (2011) when carrot was grown, no infestation was observed. Moreover, in 2012, when tomato was grown again in the same field for experimental purposes, heavy infestation was observed (data not shown). Similarly, in another intensively sampled field where vetch (*Vicia sativa*) had been planted prior to tomato, different spatial parasitism patterns were observed in the two crops (data not shown). Additionally, Román (2013) hypothesized that different fractions of a *P. aegyptiaca* population would parasitize different crops due to high specificity between the host and the parasite. Our results, which are based on field data, suggest that the specificity could be associated with the concentration of each individual stimulant in a group of stimulants rather than to the concentration of a particular individual stimulant, as was reported by Fernandez-Aparicio et al. (2011). Specificity can also be a result of compatibility/incompatibility between the host and the parasite. However, in the test cases described above, both vetch and tomatoes were parasitized by *P. aegyptiaca*. The distribution patterns of *P. aegyptiaca* seeds that parasitized vetch and tomato might have been different due to different responses to the germination stimulants and strigolactones.

The results from the historical data demonstrated the importance of the internal infestation source and the conditions for within-field *P. aegyptiaca* spread in tomato fields. In comparison, the results from the geostatistics and spatial analysis indicate that external sources are responsible for the initial infestation in a field or in a region. The clustered spatial pattern of infestation, which was observed in 40% of the intensively sampled fields, and the fact that the clusters were found adjacent to the fields’ borders, indicates that the initial source of infestation is external. Various external sources of infestation have been suggested in the past, e.g., non-sanitized machinery, containers and compost; water runoff; and infested tomato seedlings (Goldwasser and Rodenburg, 2013). The ecoinformatics analysis together with the spatial patterns analysis raises the possibility that infested neighboring fields play a major role in the spread of *P. aegyptiaca* between fields. Additionally, a high correlation was found between infestation level and distance from infested neighboring tomato fields, in fields with no internal infestation source (**Figures 9**, **10**). In light of these results, we conducted an experiment designed to test the above premise; the experiment demonstrated that during harvest, seeds can be blown to a distance of at least 90 m (**Figure 11**) and initiate infestation in new fields that have no internal seed bank. The experiment, however, did not include “non-harvest control” i.e., cards were not placed in proximity to infested tomato fields in times when no harvest took place to explore the possibility of merely passive wind-assisted seed movement. Our interpretation about the major role the harvest operation has on *P. aegyptiaca* seed dispersal may be supported by the results of Berner et al. (1994) and van Delft et al. (1997). They both indicated that distribution of seeds of the parasitic plant *S. hermonthica* by wind was not extensive. In their studies, the maximum distance that seeds were caught was 8–12 m away from highly infested fields. Additionally, *P. aegyptiaca* shoots range between 10 and 20 cm in height, allowing seed dispersal to only a few meters (Winkler, 2009).

The simulation model results showed how *P. aegyptiaca* would probably spread in a new tomato-growing region. The model showed that if tomatoes become a major crop and are intensively grown in a new region, *P. aegyptiaca* would spread to all the fields in that region within a single decade. These dynamics are predicted under the assumptions that the crop rotation includes a tomato crop that is planted every 5 years, and that un-sanitized machinery, containers or compost are used. When the simulation results for infestation spread are compared with the actual spread that took place in the Bet-She’an Valley during the period from 2000 to 2010 (10 years), the comparison reveals that the simulation assumptions suffered only from an over-estimation. On one hand, in both Bet-She’an Valley and the simulation, the regional infestation level increased from low to medium over the ten-year periods. On the other hand, after ten years, the infestation levels were 1.52 ± 1.03 (32 fields) and 2.2 ± 0.45 (eight fields) in the Bet-She’an valley and in the virtual region, respectively. The average difference of 0.65 was statistically significant (Kruskal–Wallis test followed by the Steel–Dwass test; *p* < 0.05), and probably derives from the high intensity of the tomato crop in the very small virtual region, which does not represent real conditions. Nonetheless, the simulation accurately reflects the spread process and mechanism that are proposed, based on the study’s results.

### Ways to Minimize the Spread and Improve Control Measures

The results of this study emphasize the major effect of the agro-techniques adopted by farmers on the spread of *P. aegyptiaca* infestation. Goldwasser and Rodenburg (2013) have proposed several methods to prevent *P. aegyptiaca* spreading from internal and external sources. The main point that they put forward was the necessity of preventing seed delivery from external sources, so as to maintain a low seed bank. Sanitation can be a key factor in minimizing the spread rate. However, sanitation cannot prevent the movement of seeds from neighboring fields during harvest. A decision support system (DSS), known as PICKIT, proposed early treatments with sulfosulfuron to prevent the damage caused by *P. aegyptiaca*, together with treatments to prevent late parasitism and seed ripening of the parasite, via a foliar application of the herbicide Imazapic. This late treatment sterilizes the *P. aegyptiaca* inflorescences, and should be applied to prevent internal or external infestation (Eizenberg et al., 2012a). Another way to minimize the movement of seeds during harvest is to develop appropriate design adjustments to the combine harvester. Finally, based on the results of this study, the adverse impact of herbicides may be reduced if a site specific parasitic weed management is adopted in combination with thermal time models. For example, if a field adjacent to a neighboring field that was parasitized in the past is planted for the first time with a tomato crop, herbicides should be applied at the right time (according to the thermal model) merely along the common border with the infested field. Additionally, the history of the fields can be used to define rationale within-field infestation sampling to map the infestation pattern that could then be used to direct the application of site-specific herbicides. For example, fields with a history of infestation in tomatoes may require a few samples to characterize their infestation spatial pattern, while fields with a history of infestation in other hosts may require a much denser sampling grid. Fields with tomato crops for the first time would require dense sampling grids along a common border with an infested tomato field, and reduced sampling points towards the center of the field. Indeed, within-field sampling necessitates quick and low-cost infestation sampling techniques. In conclusion, this study showed the undesirable effects of crop rotation, infestation history and proximity to infested tomato fields on the spread of broomrape species in a commercial scale. The study combined ecoinformatics, spatial analysis, geostatistics, and designated field experiment to quantify these effects and to assist with defining the spread mechanism of the *P. aegyptiaca* between growing regions and fields.

## Author Contributions

YC contributed to the conception and design of the study, to data acquisition and data analysis, to the interpretation of the results and to all stages of the paper writing. IR conducted his MA research on this topic. He was responsible for the data collection and contributed to the data analysis and interpretation. LB and EG assisted with the statistical analysis and description. HE contributed to the conception and design of the study, to the interpretation of the results and to all stages of the paper writing.

## Conflict of Interest Statement

The authors declare that the research was conducted in the absence of any commercial or financial relationships that could be construed as a potential conflict of interest.
